# Kansas

**Published:** 1895-05

**Authors:** 


### Kansas.

The loss of a State Veterinarian as an official of the State of Kansas has resulted solely from unsatisfactory results obtained through the employment of members of this calling, during the past two or three years. In fact, so flagrant became the enormous expense piled up by one of the recent occupants of this position that it became a State issue of great force in the last campaign throughout this State. The position has become one thoroughly political in character, and whenever this condition ensues it is bound to result in disaster to the profession. Not until all these positions are made upon the basis of merit of the applicant will they become secure and the States creating and appointing them reap the reward they are entitled to through the valuable services that can and will be rendered to any State in the just recognition of the profession.

The loss of the State Veterinarian in this State was filled by the maintenance of the State Sanitary Live Stock Commission, consisting of two Republicans and one Democrat, and at present most of the investigations are conducted by one or more of this Board. They only employ a veterinarian when they deem it necessary.

